# The impact of phages on interspecific competition in experimental populations of bacteria

**DOI:** 10.1186/1472-6785-6-19

**Published:** 2006-12-13

**Authors:** Michael A Brockhurst, Andrew Fenton, Barrie Roulston, Paul B Rainey

**Affiliations:** 1 Department of Plant Sciences, University of Oxford, Oxford, OX1 3RB, UK.; 2 School of Biological Sciences, Biosciences Building, University of Liverpool, Crown Street, Liverpool L69 7ZB, UK.; 3 Laboratory of Neurobiology, Wellcome Department of Cognitive Neurology, University College London, Gower Street, London WC1E 6BT, UK.; 4 School of Biological Sciences, University of Auckland, Private Bag 92019, Auckland, New Zealand.

## Abstract

**Background:**

Phages are thought to play a crucial role in the maintenance of diversity in natural bacterial communities. Theory suggests that phages impose density dependent regulation on bacterial populations, preventing competitive dominants from excluding less competitive species. To test this, we constructed experimental communities containing two bacterial species (*Pseudomonas fluorescens *and *Pseudomonas aeruginosa*) and their phage parasites. Communities were propagated at two environmental temperatures that reversed the outcome of competition in the absence of phage.

**Results:**

The evenness of coexistence was enhanced in the presence of a phage infecting the superior competitor and in the presence of phage infecting both competitors. This occurred because phage altered the balance of competitive interactions through reductions in density of the superior competitor, allowing concomitant increases in density of the weaker competitor. However, even coexistence was not equally stable at the two environmental temperatures.

**Conclusion:**

Phage can alter competitive interactions between bacterial species in a way that is consistent with the maintenance of coexistence. However, the stability of coexistence is likely to depend upon the nature of the constituent bacteria-bacteriophage interactions and environmental conditions.

## Background

Understanding the mechanisms of species coexistence and hence the maintenance of diversity is a central theme of ecology and evolution. A number of ecological mechanisms have been suggested that allow coexistence of interspecific competitors that would otherwise competitively exclude [1]. One of these is natural enemies, such as predators, parasites and pathogens. Parasites are thought to play a key role in structuring host communities [2]; the role of shared generalist parasites in mediating competitive interactions between two or more hosts have been explored theoretically and empirically for cases where the hosts compete directly (parasite mediated competition: [3-7]) and where they do not (apparent competition: [8,9]. However, this body of work typically shows that such generalist parasites tend to result in inhibitory effects between the host species, reducing the likelihood of species coexistence, unless certain criteria are met [2,10,11]). However, many parasites are highly specific in their interactions with hosts and the role such parasites play in shaping ecological communities and maintaining host diversity has not been fully explored. In particular, specific parasites introduce an extrinsically generated source of density-dependent regulation of their host population, potentially altering the outcome of interspecific competition between host species. Such a role for specialist parasites has been hypothesised to maintain diversity in species rich tropical tree communities (Janzen-Connell hypothesis) [12,13].

The effect of specific parasites on host coexistence is particularly relevant to phage-bacteria associations, where lytic phage typically only infect one species or even a single strain of bacterium. Highly diverse communities of bacteria are found in a wide range of habitats including soil [14], aquatic, and aquatic sediment environments [15], and recent investigation indicates that this diversity is crucial to the functioning of these communities [16]. The discovery that environmental phage abundances far exceed expectations [17,18] has led to the suggestion that phage may play a role in the maintenance of diversity in natural bacterial communities [19]. Theoretical studies suggest that phage provide density dependent regulation of competitively dominant bacterial species thus preventing the exclusion of less competitive species ("Killing the winner" hypothesis [20,21]).

Empirical tests of the "killing the winner" hypothesis with bacteria and phage are limited [22]. Several studies have investigated the role of phage in mediating community composition in the laboratory. Here, the phage either had little effect on coexistence [23,24] or led to the exclusion of the parasitized competitor [23]. Indeed, these studies were not specifically designed to investigate the role of phage in promoting coexistence; an ideal experiment would study the effects of phage on coexistence in communities where one bacterial species is competitively dominant in the absence of phage. To investigate this we constructed simple communities containing two bacterial competitors, *Pseudomonas fluorescens *and *Pseudomonas aeruginosa*. The outcome of competition in mixed communities is temperature dependent, favouring *P. fluorescens *at 14°C and *P. aeruginosa *at 28°C. Both bacteria can be readily isolated from environmental sources [25] and these temperatures are well within the ambient range. We propagated replicate communities in the absence and presence of their respective viral parasites, phage SBW25Φ2 and phage PP7, both individually and in combination. Communities were propagated in experimental microcosms for 8 days and destructively sampled every 24 hours to determine the relative abundance of the bacterial competitors. We hypothesised that phage would promote more equitable coexistence of bacterial competitors by weakening competitive interactions through reductions in density of the competitively dominant species.

## Results

**Figure 1:**
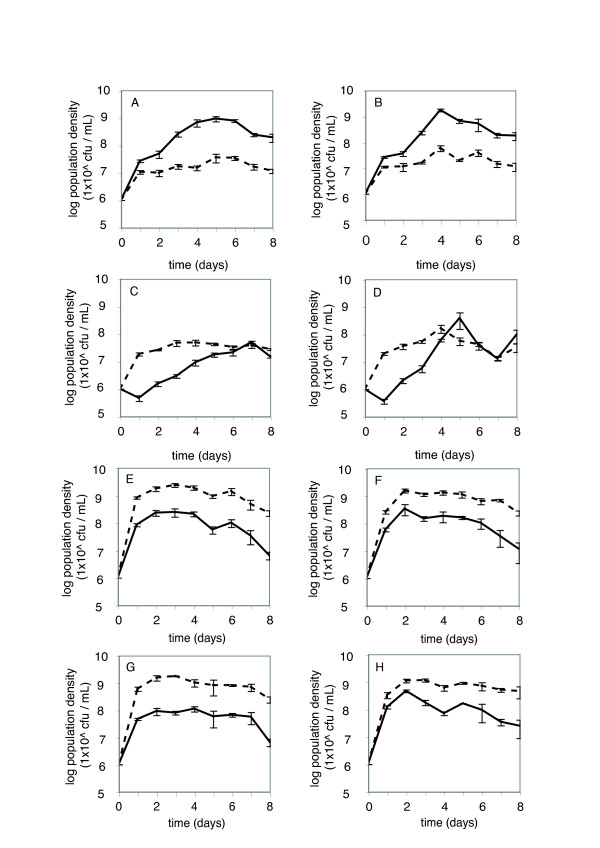
**Bacterial density through time**. Lines represent mean log population density ± SE of *P. fluorescens *(solid) and *P. aeruginosa *(dashed) at 14°C (A-D) and 28°C (E-H) in the presence of no phage (A & E), phage PP7 (B & F), phage SBW25Φ2 (C & G) and both phage (D & H).

Both bacterial species persisted for the duration of the experiment in all populations, but the relative abundance of the species varied between treatments and environmental temperatures (Figure 1). Both phage species also persisted for the duration of the experiment in all population where initially present (see methods, data not shown). In the absence of phages populations were dominated by either *P. fluorescens *at 14°C or *P. aeruginosa *at 28°C (Figure 1 panels a & e). The relative abundance of the two bacterial species was quantified using the complement of the Simpson Diversity Index (see methods) which takes into account both species richness, which was equal for all populations (i.e., 2), and the evenness of species abundance, which varied between populations. Greatest evenness was achieved when the coexisting bacterial species were at equal abundance and results in a maximal Simpson Diversity Index of 0.5.

**Figure 2:**
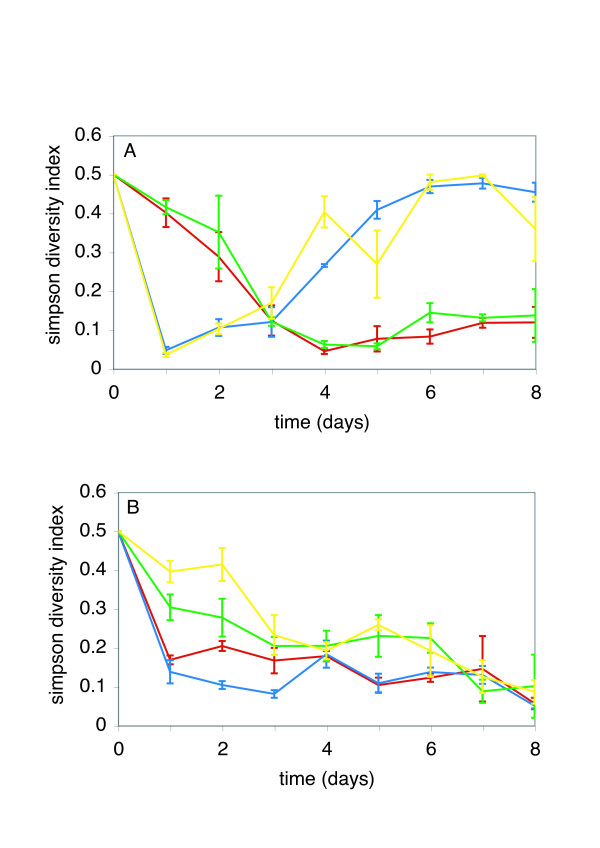
**Evenness of bacterial coexistence through time**. Lines represent mean diversity measured as the complement of the Simpson Index ± SE at 14°C (A) and 28°C (B) for communities with no phage (red), phage SBW25Φ2 (blue), phage PP7 (green) and both phage (yellow). The complement of the Simpson Index is a useful measure as it takes into account both species richness and evenness. Bacterial species richness did not vary between populations (i.e., 2 species); therefore changes in the Simpson Index here represent changes in the evenness of the community. In a community of two competing species, high values (0.5) indicate that the two species are coexisting equitably, as the community becomes increasingly dominated by a single species, so this measure tends towards 0.

At 14°C, evenness varied through time (Figure 2a; *F*_1,91 _= 4.929, *P *= 0.029) but was significantly greater in the presence of phage infecting the superior competitor (*F*_1,91 _= 16.182, *P *< 0.001), and phage infecting both competitors (Figure 2a). However, phage infecting the weaker competitor had no significant effect (*F*_1,91 _= 0.073, *P *> 0.7) and no significant interaction was observed (*F*_1,91 _= 0.158, *P *> 0.6). The population density of the superior competitor was significantly reduced by its phage (Figure 1a–d; *F*_1,91 _= 154.306, *P *< 0.001); concomitantly, population density of the weaker competitor was increased in the presence of phage infecting the superior competitor (*F*_1,91 _= 35.505, *P *< 0.001). There was no significant effect of phage infecting the weaker competitor on population density of the weaker competitor (*F *_1,91 _= 1.121, *P *> 0.2) and no significant interactive effect of both phage species being present was observed (superior competitor density, *F*_1,91 _= 2.328, *P *> 0.1; weaker competitor density, *F*_1,91 _= 0.044, *P *> 0.8).

At 28°C, evenness varied through time (Figure 2b; *F*_1,90 _= 55.951, *P *< 0.001), but was significantly greater in the presence of phage infecting the superior competitor (*F*_1,90 _= 45.254, *P *< 0.001), and phage infecting both competitors (Figure 2b). However, this effect was only apparent for the first few days of the experiment and evenness declined to low levels in all populations by the end of the experiment (Figure 2b). Phage infecting the weaker competitor had no significant effect on evenness (*F*_1,90 _= 0.014, *P *> 0.9), however there was a significant interactive effect on evenness of both phage species being present (*F*_1,90 _= 4.331, *P *= 0.04). While a marginally insignificant reduction in the population density of the superior competitor by its phage was observed (Figure 1e–h; *F*_1,90 _= 3.381, *P *= 0.069), the population density of the weaker competitor was significantly increased in the presence of phage infecting the superior competitor (*F*_1,90 _= 4.484, *P *= 0.037). There was no significant effect of phage infecting the weaker competitor on population density of the weaker competitor (*F*_1,90 _= 0.866, *P *> 0.3) and no significant interaction was observed (superior competitor density, *F*_1,90 _= 0.344, *P *> 0.5; weaker competitor density, *F*_1,90 _= 2.439, *P *> 0.1).

Total productivity, measured as the combined densities of both bacterial species, was significantly greater at 28°C compared to 14°C (*t*-test of total bacterial densities averaged through time; *T *= 6.518, d.f. = 11; *P *< 0.0001).

## Discussion

These results provide equivocal empirical support for the hypothesis that phage play a role in shaping bacterial community composition by promoting maintenance of bacterial coexistence [20,21]. While all populations contained both bacterial species for the duration of the experiment (i.e., equal species richness), coexistence was more equitable in populations containing phage infecting the superior competitor (i.e., greater evenness). More even communities are likely to be less susceptible to stochastic species loss and display greater ecosystem functioning [26]. As predicted by theory [20,21], the results indicate that the mechanism responsible was the weakening of competitive interactions through phage induced reductions in population density of the superior competitor that allowed concomitant increases in population density of the weaker competitor. Crucially, this occurred both in the presence of phage infecting the superior competitor, and in the presence of specific phages infecting both competitors. This is in line with ecological theory, which, due to the density dependent feedback between host density and parasite abundance, predicts that the lower the population density of hosts, the smaller the impact of the parasite on host density [27]. We show that this acts to broaden the conditions favouring coexistence. Such conditions, where both strong and weak competitors are parasitized, are likely to be the case in many natural environments where phage diversity and abundance is as high if not higher than that of prokaryotes [22].

However, patterns of evenness of coexistence through time in the presence of phage infecting the superior competitor were markedly different at the two environmental temperatures. While evenness of coexistence plateaued at relatively high and equitable levels at 14°C, evenness declined through time and was unstable at 28°C. There are two possible, not mutually exclusive, explanations for this. First, it is possible that the higher productivity of the communities at 28°C may have destabilised the evenness of coexistence by supporting higher population densities and thereby strengthening competitive interactions. The potentially destabilising effect of increased productivity upon the coexistence of competitors is well known [28]. Empirical studies with populations of *Escherichia coli *and bacteriophage T-2 have shown that increased productivity can alter the relative importance of competition and parasites in determining community composition [29]. Low productivity environments favoured highly competitive phage-sensitive *E. coli*, while high productivity environments favoured less competitive resistant *E. coli*; hence competition was more important at low productivity and parasites were more important at high productivity. Our results suggest that for more complex multi-species communities this may not always be the case. Here, competitive interactions in the presence of phage were stronger and coexistence less even in the more productive 28°C communities because densities were higher.

Second, this effect may have been due to the far weaker effect of phage PP7 on *P. aeruginosa *population density at 28°C, compared to the effect of phage Φ2 on *P. fluorescens *density at 14°C (Figure 2); hence phage altered competitive interactions less strongly at 28°C. The unequal effects of the two phage species suggests that they may be interacting with their bacterial hosts in different ways. The persistence of both phage species throughout the experiment suggests that this was not due to differential denaturing at the different temperatures. However, his may have resulted from species-specific ecological effects, such as one species producing more phage particles per infection, resulting in greater phage production. In addition, previous studies have shown that *P. fluorescens *and Φ2 undergo persistent antagonistic coevolution (reciprocal adaptation of host defence and parasite counter-defence) [30-32], whereas no such coevolution has been observed between PP7 and *P. aeruginosa *[33]; here phage persist on a subpopulation of susceptible hosts that is maintained because of the cost of resistance [34]. In nature however, phage diversity and abundance is likely to outstrip that of the bacterial community [22], thus each bacterial competitor may be interacting with a consortia of infectious phage rather than a single species. Bacteria will also be exposed to other consumers such as metazoan predators and grazers. Under these circumstances the mismatched effects of individual interactions may less significant. Indeed, in communities of herbivores and their predators, higher consumer diversity was associated with greater suppression of herbivore populations [35], suggesting that diverse consumer communities may be able to suppress competitively dominant bacterial species across a range of environmental conditions.

It should be noted that the results of this study might be somewhat dependent upon the culture conditions employed. Batch culture as used here results in depletion of resources through time and can been considered analogous to seasonality [36]. This imposed a limit on the duration of these experiments. An alternative approach would have been to employ chemostats, where constant environmental conditions can be maintained. This would have had the advantage that experiments could have been extended over much greater timescales than those reported here. As such these results may be more relevant to seasonal systems where there is a periodic influx of nutrients rather than those with relatively constant nutrient supply.

These results highlight that both biotic and abiotic factors can play an important role in determining species coexistence. In line with a number of classic studies, we show that the outcome of interspecific competition can be altered by environmental temperature (reviewed in [37]). In this study, the outcome of competition between *P. fluorescens *and *P. aeruginosa *in the absence of phages was reversed by increasing environmental temperature from 14°C to 28°C. In addition, the diversity maintaining effect of phages was much weaker at the higher environmental temperature. This, along with other studies [38,39], may have implications for diversity maintenance in natural populations as global temperatures increase, suggesting that existing mechanisms that maintain diversity may no longer function under changed abiotic conditions.

Previous studies have shown that phage can drive diversification within and between bacterial populations [34,40-42], here we show that phage may also play a role in shaping bacterial community composition. In line with other studies of both laboratory and field communities, these results suggest that phage can act to regulate bacterial density and alter the relative abundance of bacterial species within a community [19,22,43,44]. However, our results also suggest that the effects of phage can be dependent upon the constituent species and environmental conditions. Bacteria perform a range of ecosystem processes [45,46] and it is likely that diversity is crucial to the functioning of bacterial communities [16]. As hypothesised [19] the co-occurrence of abundant phage populations with diverse bacterial populations [17,18,22] may indeed be essential to the maintenance of such diversity.

## Conclusion

In support of recent theory [20,21], these results suggest that phage can alter competitive interactions between bacterial species in a way that is consistent with the maintenance of coexistence. However, the stability of coexistence is likely to depend upon the nature of the constituent bacteria-bacteriophage interactions and environmental conditions. This has implications for our understanding of the maintenance of bacterial diversity which is thought to be crucial to the functioning of a range of ecosystem processes [16,45,46].

## Methods

### a. Culturing and assays

192 microcosms (30 ml glass universal bottles containing 6 ml of KB media supplemented with 0.0024% pantothenic acid) were inoculated at a 1:1 ratio with 10^6 ^cells of both *Pseudomonas aeruginosa *PAO1 and *Pseudomonas fluorescens *SBW25 *panB *(a neutral marked strain that requires an exogenous source of pantothenic acid [47]) that had been previously grown for 24 hours at 28°C in an orbital shaker at 200 rpm. One quarter of the microcosms were then immediately inoculated with 10^7 ^particles of phage PP7, one quarter were inoculated with 10^7 ^particles of phage SBW25Φ2, one quarter were inoculated with 10^7 ^particles of both phages PP7 and SBW25Φ2, and finally one quarter were inoculated with no phage. One half of the microcosms in each treatment were then propagated in a static incubator at 14°C, the other half were propagated in a static incubator at 28°C. Twenty-four populations (three replicate microcosms from each phage treatment at both temperatures) were destructively sampled each day for eight days. Samples of each population were stored in 20% glycerol at -80°C. Population densities of bacteria were assayed by counting colonies grown on vitamin-free KB agar supplemented with 4.8 × 10^-6 ^% pantothenic acid. On this medium the pantothenate marked strain is readily distinguished by its greatly reduced size. Phage were isolated from bacteria by vortexing a sample of each population in 10% chloroform and centrifuging to remove bacterial debris, leaving phage in the supernatant. Phage population densities were not measured, but phage presence was assayed each day by plating a drop of supernatant on an agar plate with a soft agar top-layer containing exponentially growing *P. fluorescens *or *P. aeruginosa*. Phage persisted for the duration of the experiment in all populations and no cross-contamination was observed (data not shown). As a measure of the evenness of coexistence we used a diversity index: this measured by determining the species of 100 random colonies on vitamin-free KB agar supplemented with 4.8 × 10^-6 ^% pantothenic acid, and calculated as the complement of Simpson's index of concentration [48]:

1−λ=(1−∑ipi2)(NN−1)
 MathType@MTEF@5@5@+=feaafiart1ev1aaatCvAUfKttLearuWrP9MDH5MBPbIqV92AaeXatLxBI9gBamXvP5wqSXMqHnxAJn0BKvguHDwzZbqegyvzYrwyUfgarqqtubsr4rNCHbGeaGqipGI8VfYxH8qipiYdHaVhbbf9v8qqaqFr0xc9pk0xbba9q8WqFfeaY=biLkVcLq=JHqVepeea0=as0db9vqpepesP0xe9Fve9Fve9GapdbaqaaeGacaGaaiaabeqaamqadiabaaGcbaGaeGymaeJaeyOeI0Iaeq4UdWMaeyypa0ZaaeWaaeaacqaIXaqmcqGHsisldaaeqbqaaiabdchaWnaaDaaaleaacqWGPbqAaeaacqaIYaGmaaaabaGaemyAaKgabeqdcqGHris5aaGccaGLOaGaayzkaaWaaeWaaeaadaWcaaqaaiabd6eaobqaaiabd6eaojabgkHiTiabigdaXaaaaiaawIcacaGLPaaaaaa@4C4D@

where *p*_*i *_is the proportion of the *i*th morph and *N *is the total number of colonies sampled. This measure is the probability that two randomly selected colonies are different species. The Simpson Index is a useful measure of the evenness of coexistence; in a two-species community, high values (0.5) indicate that the two species are coexisting equitably, as the community becomes increasingly dominated by a single species, so this measure tends towards 0.

### b. Statistical analyses

Log transformed population density of each bacterial species and coexistence at 14°C and 28°C were analysed in separate ANCOVAs with time as a covariate and phages PP7 and Φ2 as factors. Measures of density and coexistence at time-point zero were excluded from analyses.

## Authors' contributions

Experiments conceived and performed by MAB and PBR, data analysed by MAB and BR, manuscript written by MAB, AF and PBR.
